# A pilot study to delimit tsetse target populations in Zimbabwe

**DOI:** 10.1371/journal.pntd.0005566

**Published:** 2017-05-03

**Authors:** Gerald Chikowore, Ahmadou H. Dicko, Peter Chinwada, Moses Zimba, William Shereni, François Roger, Jérémy Bouyer, Laure Guerrini

**Affiliations:** 1 Tsetse Control Division, Department of Livestock and Veterinary Services, Ministry of Agriculture, Mechanisation and Irrigation Development, Harare, Zimbabwe; 2 Centre de Coopération Internationale en Recherche Agronomique pour le Développement, UMR ASTRE CIRAD-INRA « AnimalS, health, Territories, Risks and Ecosystems », Campus international de Baillarguet, Montpellier, France; 3 Department of Biological Sciences, University of Zimbabwe, Harare, Zimbabwe; 4 Unité Mixte de Recherche Interactions hôtes-vecteurs-parasites-environnement dans les maladies tropicales négligées dues aux trypanosomatides, Centre de Coopération Internationale en Recherche Agronomique pour le Développement (CIRAD), Montpellier, France; 5 Centre de Coopération Internationale en Recherche Agronomique pour le Développement, Unité de Recherche Animal et Gestion Intégré des Risques, Research Platform Production and Conservation in Partnership, University of Zimbabwe, Harare, Zimbabwe; Institut de recherche pour le developpement, FRANCE

## Abstract

**Background:**

Tsetse (Glossina sensu stricto) are cyclical vectors of human and animal trypanosomoses, that are presently targeted by the Pan African Tsetse and Trypanosomiasis Eradication Campaign (PATTEC) coordinated by the African Union. In order to achieve effective control of tsetse, there is need to produce elaborate plans to guide intervention programmes. A model intended to aid in the planning of intervention programmes and assist a fuller understanding of tsetse distribution was applied, in a pilot study in the Masoka area, Mid-Zambezi valley in Zimbabwe, and targeting two savannah species, *Glossina morsitans morsitans* and *Glossina pallidipes*.

**Methodology/Principal findings:**

The field study was conducted between March and December 2015 in 105 sites following a standardized grid sampling frame. Presence data were used to study habitat suitability of both species based on climatic and environmental data derived from MODIS and SPOT 5 satellite images. Factors influencing distribution were studied using an Ecological Niche Factor Analysis (ENFA) whilst habitat suitability was predicted using a Maximum Entropy (MaxEnt) model at a spatial resolution of 250 m. Area Under the Curve (AUC), an indicator of model performance, was 0.89 for *G*. *m*. *morsitans* and 0.96 for *G*. *pallidipes*. We then used the predicted suitable areas to calculate the probability that flies were really absent from the grid cells where they were not captured during the study based on a probability model using a risk threshold of 0.05. Apart from grid cells where *G*. *m*. *morsitans* and *G*. *pallidipes* were captured, there was a high probability of presence in an additional 128 km^2^ and 144 km^2^ respectively.

**Conclusions/Significance:**

The modelling process promised to be useful in optimizing the outputs of presence/absence surveys, allowing the definition of tsetse infested areas with improved accuracy. The methodology proposed here can be extended to all the tsetse infested parts of Zimbabwe and may also be useful for other PATTEC national initiatives in other African countries.

## Author summary

Tse-tse flies are vectors of human and animal trypanosomoses, that are presently targeted by the Pan African Tsetse and Trypanosomiasis Eradication Campaign (PATTEC) coordinated by the African Union. In Zimbabwe, the government has devoted a full section of the veterinary services to tsetse and trypanosomosis control but the delimitation of tsetse infested areas, which is a pre-requisite to achieve effective control still requires improvement. Here we present a methodology that could help delimit target areas throughout the country, in a pilot study area located in the Masoka area, Mid-Zambezi valley in Zimbabwe, and targeting two savannah species, *Glossina morsitans morsitans* and *Glossina pallidipes*. The study, which was carried out in preparation for a vector control campaign, allowed to discriminate areas where tsetse presence was certain, likely or unlikely Habitat degradation due to agricultural activities seemed to play a pivotal role in determining the infestation by tsetse since settled areas had low probabilities for both species which was expected in this group. Application of this model will help reduce the cost of delineating tsetse infested areas in other parts of Zimbabwe and may also be useful for other PATTEC national initiatives in other African countries at a time when funding for tsetse control programmes is reduced.

## Introduction

Trypanosomosis is one of the major constraints to rural development in sub-Saharan Africa [1]. Tsetse (*Glossina* spp.), the primary vectors of animal and human trypanosomosis, are found in the semi-arid, sub-humid and humid lowlands of 37 countries across the continent with a potential distribution range of some 8.7 million km^2^[2]. This disease places approximately 50 million cattle at risk with losses amounting to US$4.75 billion annually [3].

In Zimbabwe, an area of approximately 180,000 km^2^ of the total 390,757 km^2^ was deemed to be ecologically suitable for tsetse before the rinderpest epizootic of 1896 [4]. Sustained interventions resulted in the clearance of tsetse flies from most of this area, with 50,000 km^2^ being cleared since 1980. Tsetse are now confined to approximately 28,000 km^2^ in North-Western and Northern Zimbabwe. However, tsetse transmitted trypanosomosis remains a challenge in areas close to tsetse infested areas with a total of 240 African Animal Trypanosomosis (AAT) cases being reported to the OIE between 2009 and 2015[5]. A Human African trypanosomosis (HAT) focus also exist in the Hurungwe District and Mana Pools areas in the Northern parts of the country [6]where 25 cases of the acute form of HAT caused by *Trypanosoma rhodesiense* were detected through passive surveillance between 2009 and 2015 [7]. The country has committed to eradicate tsetse and trypanosomiasis in the framework of the African Union coordinated Pan-African Tsetse and Trypanosomiasis Eradication Campaign (AU-PATTEC), a decision (AHG/156 (XXXVI)) by African Heads of State and Government during the 36th Ordinary Summit of the OAU, Lome, Togo held in July 2000. The distribution of tsetse and their abundance play an important role in the epidemiology of trypanosomosis and often forms the basis for intervention programmes. Insect intervention and pre-intervention programmes require accurate and up–to–date information on the spatial and temporal distribution of target insects[8]. Strategies to control or eventually eliminate the problem posed by trypanosomosis must rely on tsetse ecology and suitable fly distribution data [9]. However, it has been decades since the latest tsetse distribution maps at the continental level were produced [10].

A number of studies have been carried out in order to understand tsetse population dynamics and these have resulted in an increased understanding of the link between the environment and tsetse presence and abundance [11,12]. It has also been established that tsetse are highly dependent on particular habitats for their survival, therefore ecological and land use change has a major impact on fly populations and the associated disease risks [13]. The distribution, prevalence and impact of vector-borne diseases are often affected by anthropogenic environmental changes that alter interactions between the host, the parasite and the vector [14].

Recent advances in geospatial technology have enabled the development of models in the study of diseases and parasites. Georeferenced datasets and spatial analysis techniques have great potential to support the planning and implementation of interventions against human and animal diseases including African trypanosomosis [15]. Geographic Information Systems (GIS) based distribution mapping can help identify areas of occurrence at the micro-level, where species-specific, environmentally friendly control measures can be strengthened[16].

In recent years, tsetse and trypanosomosis distribution models have been developed at different scales. Distribution models have been produced at a continental scale from low spatial resolution data, using the Advanced Very High Resolution Radiometer (AVHRR) data from the NOAA (www.noaa.gov) satellite that present a spatial resolution of 28 km [17]. This level of resolution does not allow an accurate identification of suitable habitats for tsetse flies that are found diluted in the surrounding pixels[11]. On the contrary, studies conducted at a higher resolution in Senegal recently and based on Maximum Entropy (MaxEnt) models assisted in the identification of pockets of infestation that had been missed by surveys [18]. A study conducted in the North-Western parts of Zimbabwe has also shown great potential in modelling the distribution of suitable tsetse habitats, information that can be used in the planning of intervention programmes [19].

Here we propose to combine this approach (Maxent models) to probability models that has been used previously to delimit tsetse control areas and that stipulate that tsetse can still be present despite a series of zero catches [20,21]. The goal is to prepare for control operations in the study area, but also to produce a standardized method allowing optimizing the definition of tsetse infested areas within the framework of PATTEC.

## Materials and methods

### Ethics statement

The field work was authorised by the Tsetse Control and Division, Department of Livestock and Veterinary Services.

### Study area

**Fig 1:**
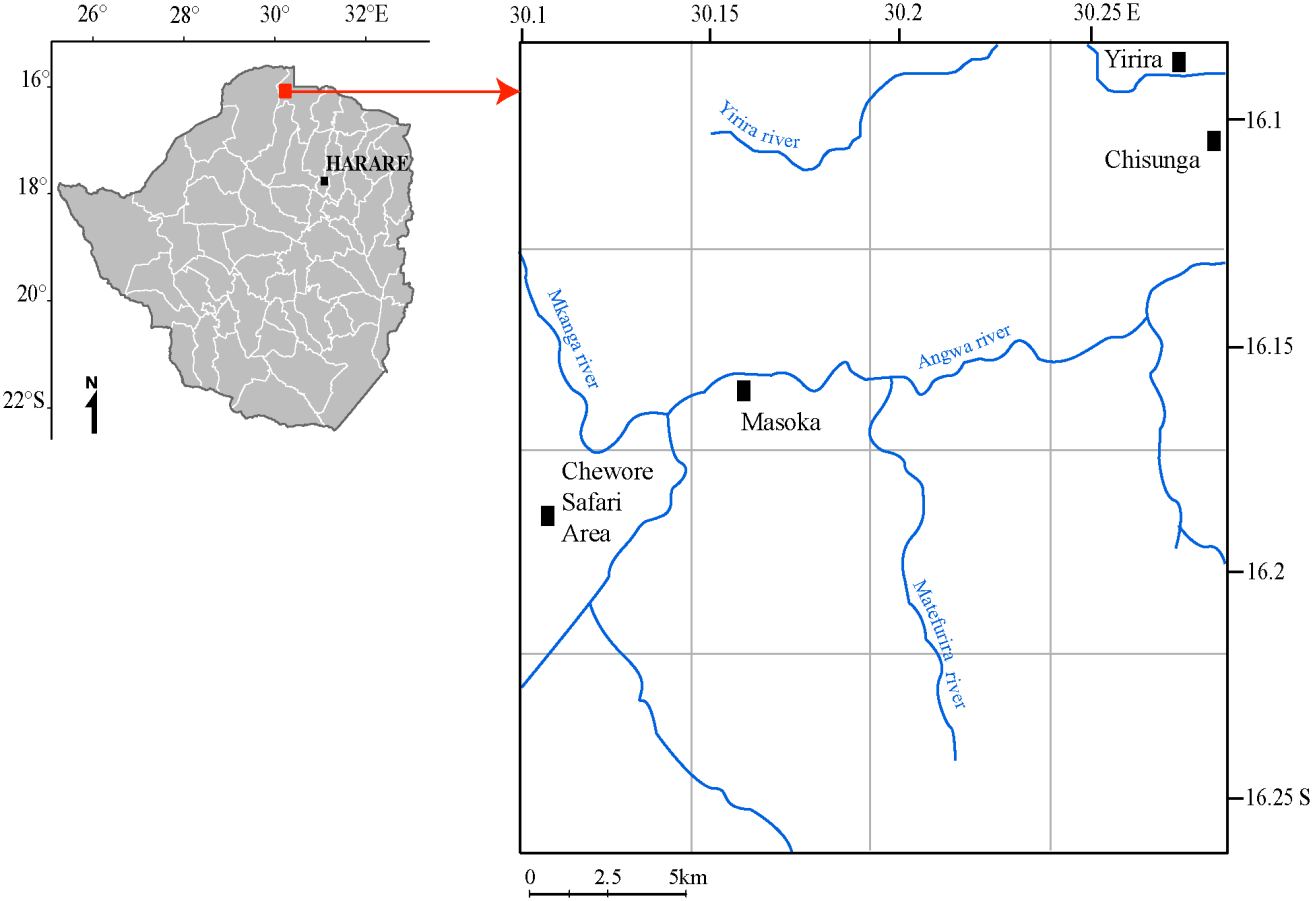
Location of the study site in the Masoka area, Mbire District, in Zimbabwe.

The study was conducted in Masoka area, Mbire District (16.00° to 16.28°S and 30.1° to 30.28°E) between March 2015 and December 2015 (Fig 1). This area belongs to the Natural Farming Region IV of Zimbabwe which receives between 650 and 800 mm of rainfall annually and is suitable for livestock and drought resistant crop production. During the dry season, most of the vegetation sheds its leaves and annual grasses and shrubs dry out. A concentration of leafy vegetation is left along water courses, although most of these are temporary. The area is part of the Community Areas Management Programme for Indigenous Resources (CAMPFIRE) scheme, which advocates for the conservation of natural resources, including wildlife. The area thus has a variety of wild animals, the most common ones being buffalo (*Syncerus caffer*), elephants (*Loxodonta africana*), warthog (*Phacochoerus africanus*), among other important tsetse hosts. The distribution of these wild hosts in the dry season is mainly influenced by water availability, as more animals were sighted towards Chewore Safari Area, a protected Parks and Wildlife Authority of Zimbabwe Estate. According to a census conducted by Zimstat the community has an estimated population of 1,632 inhabitants distributed among 300 households [22]. Agriculture is the major activity, with production centred on cattle and goat rearing, cotton and small grains production. Cattle form an important source of blood meal for tsetse, especially in areas with low wild host densities [23]. The Masoka community has a herd of 180 cattle (Division of Veterinary Services Nov 2015 Census). The tsetse population occupying this area has not been affected by intervention programmes instituted by the Division of Tsetse Control over the past 19 years [24] with control activities concentrated along Manyame River some 70 km away.

### Entomological data

Tsetse data were obtained using a grid based sampling method outlined in the Food and Agriculture Organisation (FAO)/International Atomic Energy Agency (IAEA) entomological baseline data collection manual of 2008 [25]. The study area was divided into a grid of 110 identical cells measuring 2 km × 2 km and a minimum of one and a maximum of three epsilon traps baited with sachets containing a mixture of 3-n-propyl-phenol, o-cten-3-ol and 4-methyl-phenol in the ratio of 1;4;8 [26] were placed in cells perceived to have suitable habitat. In each sampled cell, sites perceived to be suitable tsetse habitat were chosen based on a supervised classification of a SPOT 5 image. A survey team led by an experienced Tsetse Field Assistant, chose the actual site on the ground based on recommendations by Vale [12] in order to maximise on catches. Each sampling site was geo-referenced using a hand-held GPS receiver and monitoring was done after seven days. Samples were collected from 105 sites between March 2015 and December 2015. Captured flies were identified morphologically using identification keys developed by Buxton [27] and Mulligan [28] and specimens were preserved in 90% alcohol.

### Environmental data

#### Moderate Resolution Imaging Spectroradiometer (MODIS) data

MODIS (or Moderate Resolution Imaging Spectroradiometer) is a sensor on the Terra (EOS AM-1) and Aqua (EOS PM-1) satellites. The Terra satellite orbits the earth from north to south passing the equator in the morning, whilst Aqua orbits in the opposite direction passing over the equator in the afternoon. Both stellites view the entire earth's surface every 1 to 2 days, acquiring data in 36 spectral bands (http://modis.gsfc.nasa.gov). MODIS products were acquired from NASA Earth Observing System data server.

Among the various sensors and types of satellite images used, MODIS images are considered to have a good compromise between spatial and temporal resolution and this is one reason why they are widely used in epidemiological studies [29]. Satellite images spanning 10 years (January 1, 2003—January 31, 2013) from the Terra and the Aqua satellites were downloaded, cleaned and summarized into meaningful statistics (mean, minimum and maximum). We used in particular thermal and vegetation indices.

### Temperature indices

The temperature is a parameter that plays an important role in the tsetse life cycle and Land Surface Temperature is among the commonly used temperature indicators. Land Surface Temperature (LST) is calculated from the measurement of radiation emitted by the earth surface and it is highly correlated withthe air temperature [30]. 8 days daytime (DLST) and night-time LST (NLST) were extracted at 1km spatial resolution from MODIS MOD11A2/MYD11A2 temperature and emissivity products. The data werefiltered and temporally aggregated into statistics that can be used to describe the thermal profile of the study area. LST is used in many studies of species distribution and spatial epidemiology. In this study, they were used as proxies for both air and soil temperature which play an important role in tsetse habitat selection.

### Vegetation indices

Among the indices commonly used in epidemiological studies are vegetation indices, a measurement of chlorophyll activity. These indices allow the differentiation of bare ground from the vegetation and also of various vegetation types. The most commonly used is the NDVI (Normalized Difference Vegetation Index), in addition to the NDVI, other vegetation indices such as EVI (Enhanced Vegetation Index) can be used but according to Hay [31], EVI is particularly useful since it performs better than NDVI over high biomass areas. The vegetation continuous field (VCF) is also an important vegetation index that can be used to capture the density of tree cover. Regarding our study area, we used both EVI and the VCF (Treecover) to capture the effect of woody vegetation on tsetse habitat. It is also important to note that EVI and other vegetation indices have already been used several times to predict tsetse flies’ density in West Africa [11,18,32,33].

### Reflectance indices

The reflectance, in the mid-infrared is used to measure the radiation of bare soils. This index is correlated with the Land surface temperature. Luxuriant vegetation is characterized by a low MIR. With the EVI, this index allows to characterize the vegetation well as the soil temperature.

### Topographical indices

Various topographic indices such as slope, topographic wetness index (TWI) and aspect (slope direction) can be extracted from Digital Elevation Model (DEM). DEM, slope and aspect can be used to describe the elevation and exposure to the sun whereas topographic wetness index measure soil humidity. These indices were also used to model habitat suitability for the two species.

### Satellite Pour l’Observation de la Terre (SPOT 5) data

**Fig 2:**
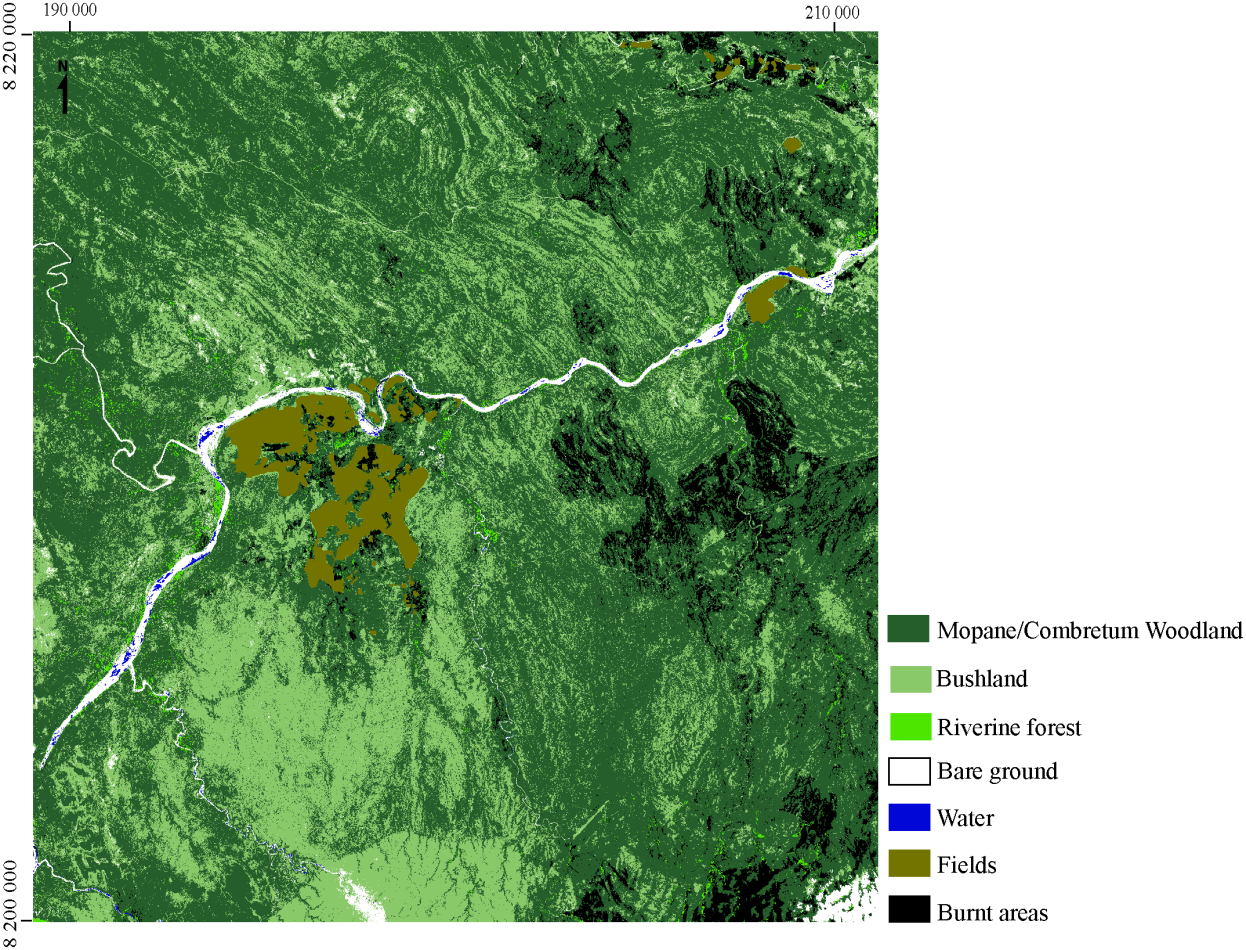
Land cover in Masoka area. Land cover units were discriminated based on a supervised classification of Spot imagery from November 2014.

A high-resolution remotely-sensed satellite image acquired on the 9th of November 2014 by the Satellite Pour l’Observation de la Terre 5 (SPOT 5 with a spatial resolution of 2.5m) was used to identify suitable areas for tsetse. A supervised classification of land cover was realized with Envi 5.1software (www.exelisvis.co.uk), based on a maximum likelihood classifier (Fig 2). Eighty three polygons were digitized manually and 106 GPS filed observations were used to validate the classification. The supervised classification was validated from the calculation of a confusion matrix and the Kappa Index of Agreement coefficient (0.95). The pair comparisons of the landcover classes gave a separability coefficient between 1.97 and 2, corresponding to an absence of confusion of the pixels allocated within each class [34]. Seven classes of land cover were identified from which 4 (mopane, riverine forest, crop field and bush land), were used as predictors in the habitat suitability models. For each of these classes, the patch density (number of patches) and the surface of patches inside the prediction pixels at a resolution of 250m were calculated. The list of the remotely-sensed data and their spatial and temporal resolution used in the present study are presented in Table 1.

**Table 1:** Description of the remote sensed environmental and climatic data used for the MaxEnt model.

Product	Name	Type	Spatial resolution (m)	Temporal resolution (day)
MOD13Q1/MYD13Q1	Ndvi	Vegetation	250 x 250	16
MOD13Q1/MYD13Q1	Mir	Thermal	250 x 250	16
MOD11A2/MYD11A2	Dlst	Thermal	250 x 250	8
MOD11A2/MYD11A2	Nlst	Thermal	250 x 250	8
Spot 5	Landcover	Vegetation	2.5 x 2.5	-
MOD13Q1/MYD13Q1	Evi	Vegetation	250 x 250	16
Slope		Topographic	250 x 250	
Twi		Topographic	250 x 250	
Aspect		Topographic	250 x 250	
Mnt		Topographic	250 x 250	

### Models

#### Exploratory analysis of habitat selection

We used a multivariate statistical method, the Ecological Niche Factor Analysis (ENFA) to characterize the habitat of the two species in the study area, using the R package adehabitatHS [35]. We did the analysis using presence data only and environmental covariates. The environmental space actually used by the species was compared with the available environmental space using two indicators, marginality and specialization. A large marginality value implies that the conditions where the species is found are far from the global environmental conditions whilst specialization measures the spread and use of the ecological space along dimensions of niche use. The higher this value, the narrower the space used by the species [36]. The species niche was summarized by an index for marginality and another for specialization, represented on a factor map within the biplot framework.

### Habitat suitability model

In the second step, we used a statistical model to predict suitable habitats for both species. We used the Maximum Entropy (MaxEnt) (www.cs.princeton.edu/~schapire/maxent), a species distribution model. MaxEnt is a machine learning algorithm that applies the principle of maximum entropy to predict the potential distribution of species from presence-only data and environmental variables. We resampled climatic and environmental data to a spatial resolution of 250 m and used them as the known features in determining the suitability index of each tsetse species within the study area. Each tsetse species, *G*. *morsitans* and *G*. *pallidipes* was modelled separately and for each we used presence data and a set of randomly generated pseudo-absence. We used leave one out cross validation (LOOCV) to compute all the model quality metrics. The model was trained n times (n = sample size) and each time we removed one observation for validation and at the end we aggregated the n metrics calculated on the validation point. The absence data were used only to assess the accuracy of each model and set a threshold for the model. We used the Receiver Operator Characteristic (ROC) curve and the associated, Area Under the Curve (AUC) as a metric for assessing the quality and performance of our prediction [37]. An AUC with values closer to 1 indicating excellent prediction. The MaxEnt software was used through its R interface in the dismo package [38].

### Probability model

#### Tsetse probability modelling

We used a probability model [20] to evaluate the probability that tsetse are actually absent in a grid cell when not sampled through a given sampling effort (number of traps/ days). We applied the model to all 2 km × 2 km grid cells were sampling was done but no tsetse were caught. The model gives the probability of observing a sequence of zero catches despite the presence of insects in the sampled area using the following formula:

P=exp(−S×t×σ ×λ )

where: *S* = number of traps deployed in the total area,

*t* = number of days for which each trap is operated,

σ = trap efficiency, and

λ = population density (number of insects/area of suitable habitat predicted using the MaxEnt models).

We calculated the probability for each grid cell using the specific number of traps, duration of trapping, and the total surface area of suitable habitat in each grid cell. The minimum number of flies in the sample area was set at 10, considering that this is an underestimation for any resident tsetse population in the absence of any control effort. The goal of this exercise was to detect resident tsetse populations and not to detect dispersing individuals. The trap efficiency, defined as the probability that a single trap catches a fly in one day given that the fly is present in an area of 1 km^2^ around the trap was defined as 0.001 for *G*. *m*. *morsitans* and 0.01 for *G*. *pallidipes* [20].

## Results

### Entomological results

**Fig 3:**
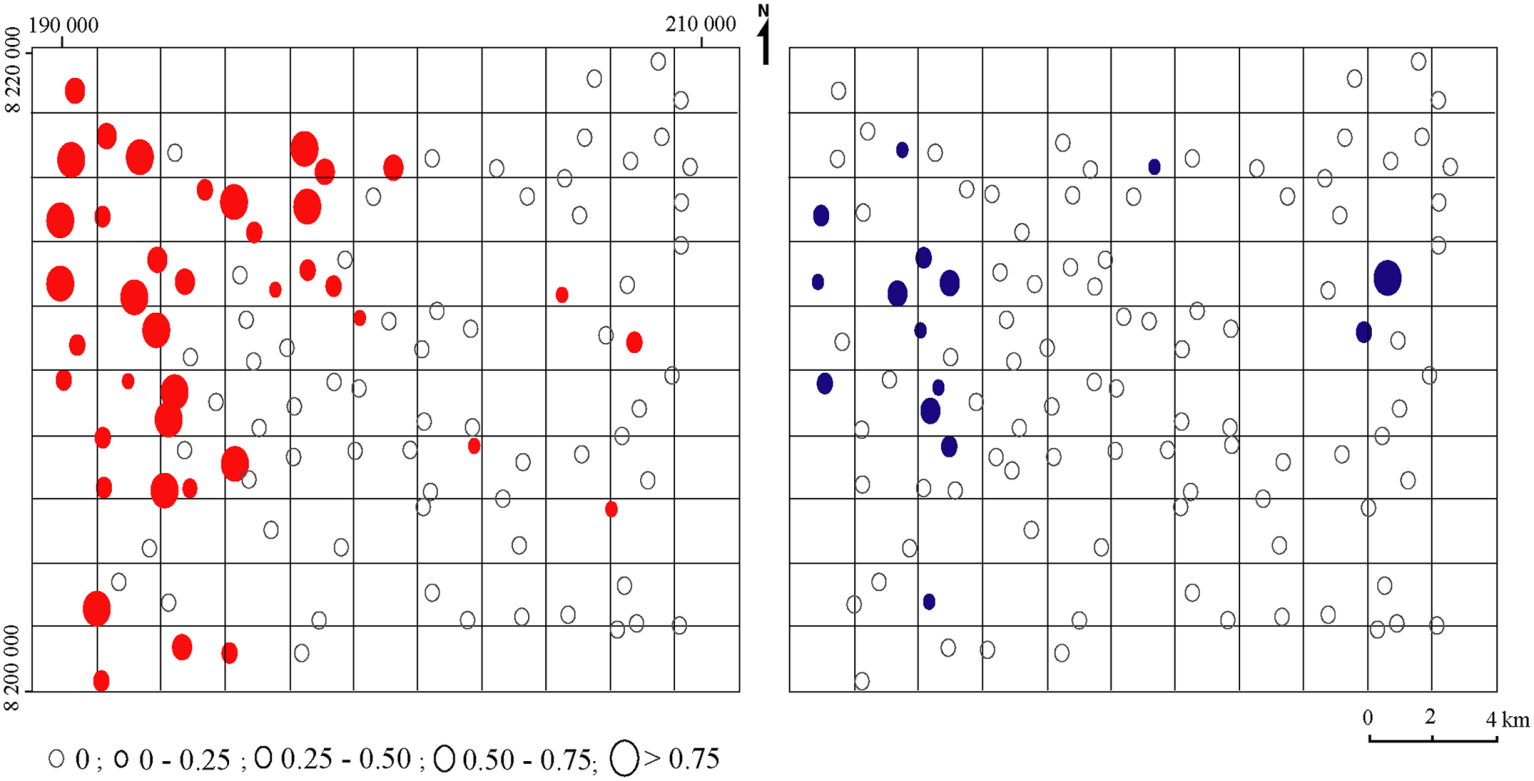
Apparent densities per trap of tsetse in the Masoka area. G. m. morsitans is presented in red color on the left and G. pallidipes in blue color on the right.

A total of 73 cells (292 km^2^) of the 110 cells were sampled with 105 traps. Survey results demonstrated a mean density of 0.27 (sd = 0.54) flies/trap/day for *G*. *m*. *morsitans*, with a presence in 40 sites distributed in 31 cells (124 km^2^) The mean density of *Glossina pallidipes* was 0.05 (sd = 0.16) flies/trap/day, with a presence in 15 trapping sites within 13 cells (52 km^2^) (Fig 3).

**Fig 4:**
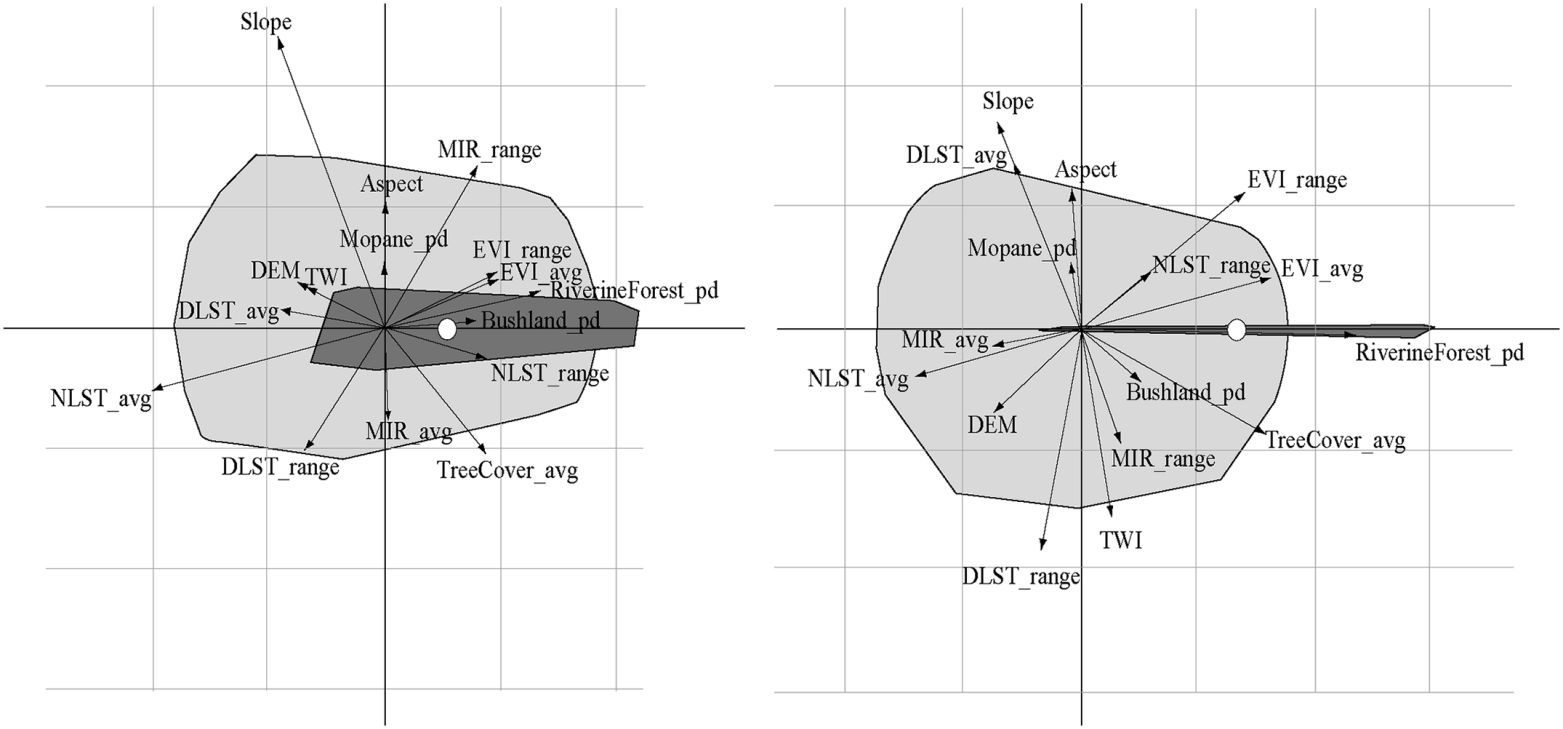
Ecological niche factor analysis (ENFA) of tsetse distribution in the Masoka area. First plans of the ENFA of *G*. *m*. *morsitans* is presented on the left panel and *G*. *pallidipes* on the right one. For each species, the light gray polygon shows the overall environmental conditions available in the study area whilst the dark gray one shows environmental conditions where each species were observed (representation of the realized niche). The small white circle corresponds to the barycenter of each species’ distribution. The first axis (marginality axis) measures the dimension in the ecological space in which the average conditions where the species lives differ from the global conditions with a large marginality value implying that the conditions where the species is found are “far” from the global environmental conditions. The second axis (specialization) measures the narrowness of the niche (ratio of the multidimensional variances of the available to occupied spaces). avg, average; pd, Patch density; NLST, Night Land Surface Temperature; DLST, Day Land Surface Temperature; DEM, Digital Elevation Model; EVI, Enhanced Vegetation Index; MIR, Mid-InfraRed; TWI, topographic wetness index.

The first plan of the ENFA showed that *G*. *m*. *morsitans* occurrence was positively correlated with vegetation indices (EVI, Riverine Forest, Average tree cover and MIR). However, most of the temperature indices exhibited a negative correlation to the species (Fig 4). Mopane woodland patch density and aspect exhibited an important influence on the habitat for the species as they were strongly correlated with the specificity axis. Average EVI accounted for most of the variance and fell outside the cloud of average conditions available in the study area.

The occurrence of *G*. *pallidipes* also showed a positive correlation to vegetation related indices with most of the temperature indices exhibiting a negative correlation. The topographic wetness index (TWI) was positively related to *G*. *pallidipes* but negatively with *G*. *m*. *morsitans* whilst night land surface temperature was the only temperature related covariate which showed a positive correlation with the occurrence of both species.

**Fig 5:**
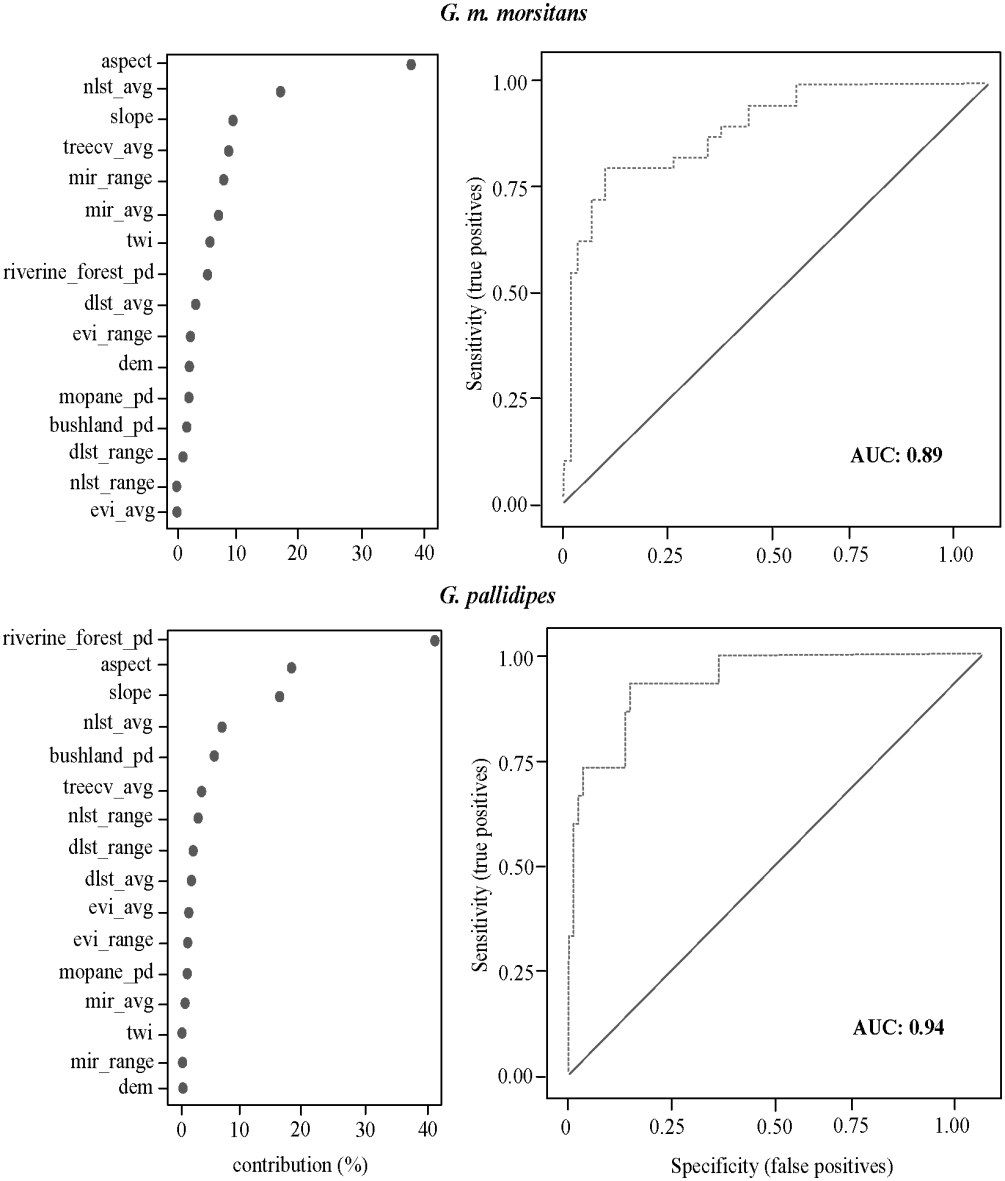
Variable contributions, ROC curves and AUCs of the Maxent models for tsetse distribution. G. m. morsitans is presented on the top pannel and G. pallidipes at the bottom. nlst, night land surface temperature; avg, average; treecv, tree cover; pd, patch density; dlst, day land surface temperature; dem, digital elevation model.

The habitat suitability models for *G*. *m*. *morsitans* and *G*. *pallidipes* had an Area Under the Curve (AUC) of 0.89 and 0.94 respectively (Fig 5). Both figures were close to one although the *G*. *pallidipes* model had a better prediction ability. However there were differences in covariates contributing to the models. The most contributive variable was “aspect” in the *G*. *m*. *morsitans* model and “riverine forest patch density” in the *G*. *pallidipes* model.

**Fig 6:**
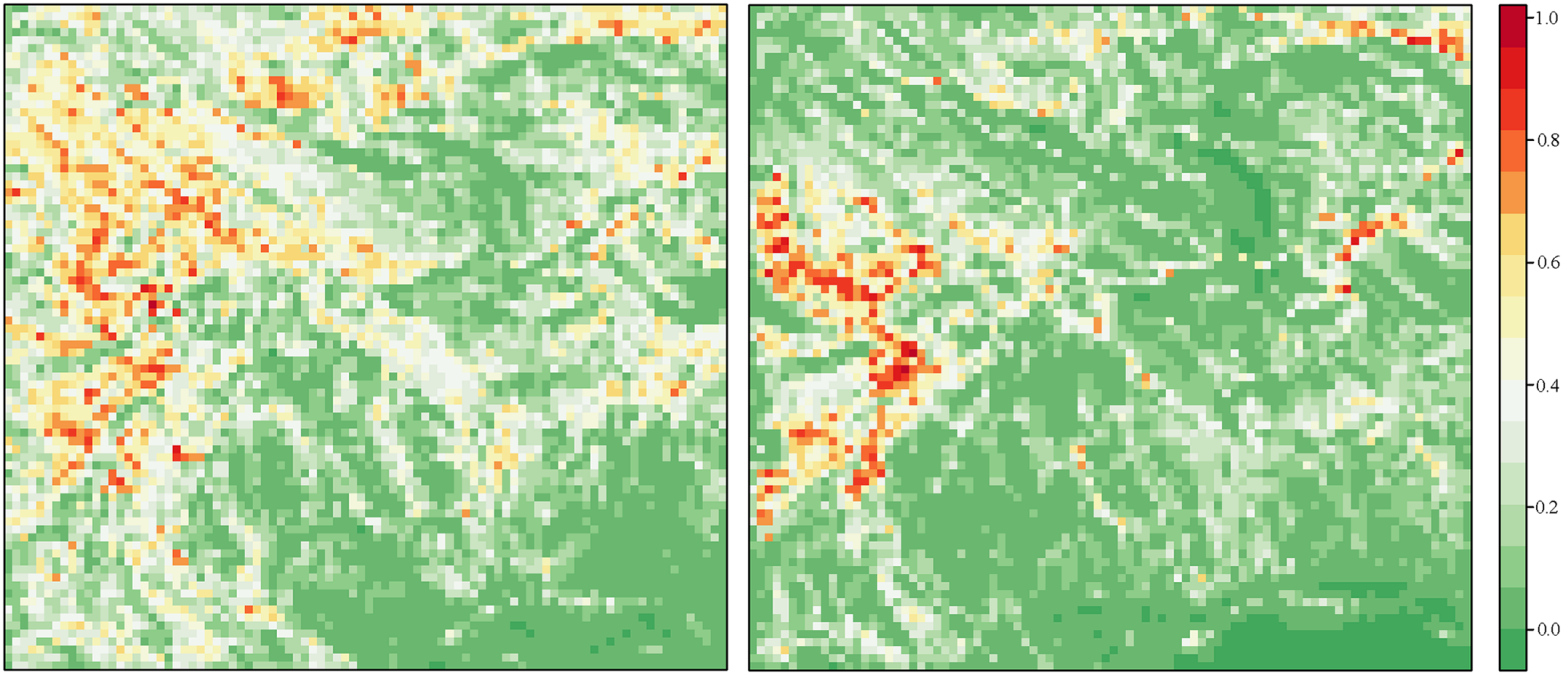
Suitable tsetse habitats predicted by the Maxent model in the Masoka area. G. m. morsitans is presented on the left panel and G. pallidipes on the right.

The resultant maps depicting habitat suitability for the species (Fig 6) show a wider area suitable for *G*. *m*. *morsitans* than *G*. *pallidipes*. There was a concentration of suitable habitat to the west of the study area which is a protected wildlife area.

### Presence probability models

**Fig 7:**
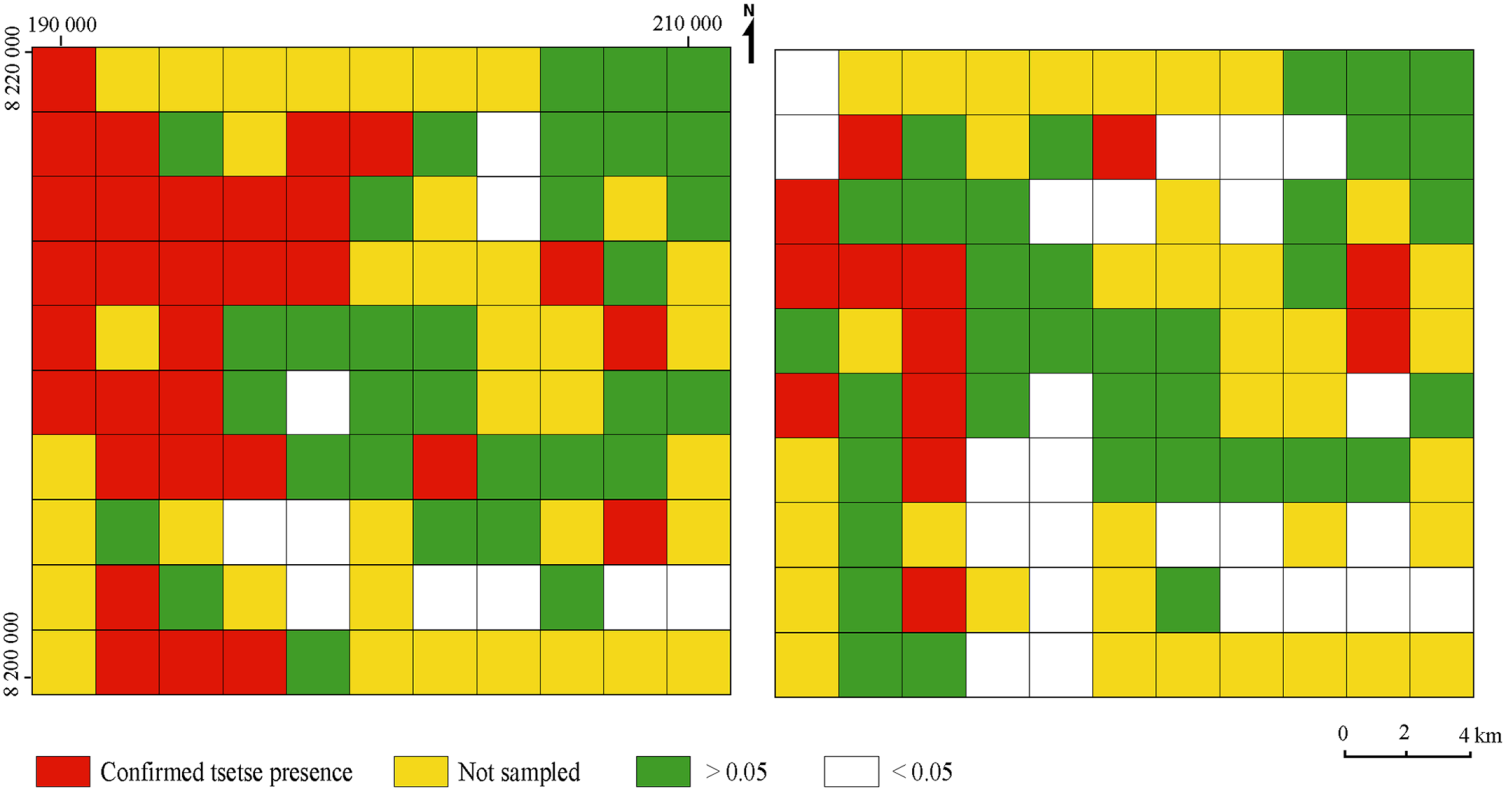
Presence probability model of tsetse in the Masoka area. G. m. morsitans is presented on the left pannel and G. pallidipes on the right. The white and green cells correspond to the probability of tsetse presence despite a sequence of zero catches.

We applied the probability model to 42 grid cells where no *G*. *m*. *morsitans* were caught. The analysis indicated a probability of *G*. *m*. *morsitans* presence below 0.05 (the level of risk accepted) in 10 grid cells where no tsetse were captured whilst 32 grid cells had a probability greater than 0.05 that *G*. *m*. *morsitans* was still present despite a sequence of zero catches. We observed the area infested with *G*. *m*. *morsitans* to be 124 km^2^ (28%) and a further 124 km^2^ (28%) had a high probability of being infested. An area of 40 km^2^ (9%) had a low probability of tsetse presence whilst the remaining 148 km^2^ (34%) were not sampled (Fig 7).

We also applied the probability model to 60 grid cells where no *G*. *pallidipes* were captured. The analysis indicated a probability of tsetse presence below 0.05 (the level of risk accepted) in 24 grid cells where no tsetse were captured whilst 36 grid cells had a probability greater than 0.05 that *G*. *pallidipes* was still present despite a sequence of zero catches. Area infested with *G*. *pallidipes* was therefore observed to be 52 km^2^ (10%), area with a low probability of tsetse presence was 96 km^2^ (22%) whilst the remaining 148 km^2^ (34%) were not sampled (Fig 7).

## Discussion

### The habitat suitability model

Habitats suitable for *G*. *m*. *morsitans* and *G*. *pallidipes* can be modelled using presence data and environmental variables [19]. This study produced habitat suitability models at a high resolution (250 m), a level which can be translated into operational plans. The habitat suitability models produced in this study had relatively large AUCs, an indication of a good predictive power. This showed that the habitat of both species, to a large extent, can be explained by the covariates used.

The two species under consideration, *G*. *m*. *morsitans* and *G*. *pallidipes* were positively correlated with vegetation indices on the first plan of the ENFA, indicating that the requirement for these covariates for these species was different than the mean conditions in the study area. The link between vegetation and tsetse has been well established through various studies [11,12]. Whilst both species were found in habitats along watercourses during the dry season, *G*. *m*. *morsitans* was also captured in deciduous woodlands of predominantly mopane trees. Studies by Vale at Rekomichi showed that there was variability in *G*. *m*. *morsitans* catches across vegetation types with seasonal effects evident whilst catches of *G*. *pallidipes* were distinctly higher in thickets than in mopane woodlands [12]. According to Cecchi *et*.*al*., deciduous woodlands and deciduous shrub-lands with sparse trees account for over 50% of the total distribution of the *morsitans* group [9].

In their model in North Western Zimbabwe, Matawa *et al*, observed that higher altitude was not associated with suitable tsetse habitat for both *G*. *m*. *morsitans* and *G*. *pallidipes* [19]. They attributed this to the effect of altitude on other climatic factors such as temperature. In our study however, the effect of altitude could not be fully explored as the elevation in the study area was more homogenous than the in North-Western Zimbabwe hence there was little variability to examine. Aspect, however, seemed to play a role in the determination of suitable habitat probably due to its association with the amount of sunlight received and subsequently temperature.

This study also demonstrated a negative correlation between suitable tsetse habitat and day land surface temperature which is a measure of air temperature. This negative correlation means *G*. *m*. *morsitans* and *G*. *pallidipes* require lower temperatures than the average values in the study area. Whilst studies on artificial refuges by Vale could not pin-point the exact temperature at which all tsetse occupy refuges, they clearly demonstrated that temperatures beyond 30°C affect tsetse [39]. Further work by Hargrove and Muzari revealed an increase in catches of male and pre-full term pregnant female tsetse in refugia at around 32°C [40]. Although no temperature measurements were made in this study, online weather sources reported episodes of maximum temperatures in excess of 40°C in the study area between October and December, values which compare well with 42.5°C observed by Hargrove and Muzari during 1998 [40]. Maximum temperature has also shown to have significant effect on tsetse survival [41] with laboratory studies showing an increase in daily mortality due to temperature [42]

The aim of modelling is to improve the quality of intervention plans leading to a reduction in costs hence accuracy of the models is of paramount importance if they are to be the basis of intervention. The distribution of tsetse hosts is a critical determinant of tsetse distribution, particularly in the *morsitans* group [13,23]. We however obtained very good predictions in the present study, probably because the density of these wild hosts is correlated with the vegetation habitats that were integrated in the prediction models.

### The probability model

This study confirmed that the absence of tsetse catches in traps does not imply absence in a locality [12,21]. Unlike other probability models build using vegetation only [21] this study used the habitat suitability model, a factor which captures major characteristics of the habitat thus increasing the robustness of the model. Suitable tsetse habitats are influenced by more factors other than vegetation alone thus the probability model produced in this study has got a greater chance of detecting grids with higher chances of infestation.

The probability model showed greater chances of both *G*. *m*. *morsitans* and *G*. *pallidipes* presence in wider parts of the study area than observed through surveys. A number of factors can be attributed to this result. Firstly, the absence of tsetse in traps, especially *G*. *m*. *morsitans*, could have resulted from a lower efficiency of traps in capturing the species (0.001) [20], a parameter which is linked to the behaviour of the species. Resting *G*. *m*. *morsitans* respond more to moving objects than *G*. *pallidipes* [43]. Whilst great care was taken to place traps in optimal sites, siting in itself is a factor which can influence the efficiency of traps [44]. The model however, still showed a high probability of *G*. *pallidipes* presence, contrary to catches recorded in traps which were low despite a better trapping efficiency (0.01) [20].

### Applications of the model

This new methodology is presented here for the first time and will allow a great enhancement of future tsetse sampling efforts. It has the potential to generate surface information (raster data) from point data (trap catches) thus providing operational information to guide planning and decision making. The model can be applied in planning the placement of insecticide treated targets as it is grid based and can also be applied to direct the focus of further surveys. Most remote sensing products are now freely available making the processing of data much cheaper thus helping national entities working on tsetse control programmes to make informed decisions in the judicious allocation of scarce resources (Prioritization of target areas based on assessed risk). Previous applications of probability modelling on riverine species in West Africa allowed the detection of isolated pockets of tsetse in areas which had been missed by surveys [18,45]. This study also demonstrated that information on areas not surveyed within target areas can be generated to guide the planning process. This is of importance as some areas can be difficult to access whilst at times resources may be limiting to obtain data from every location of the target area. However, the presence of predicted suitable habitats in these not sampled areas will be the basis to consider them as infested or not but this will need to be confirmed by additional sampling efforts.

Whilst local conditions may differ from place to place, we believe adoption of the methodology presented here would assist the country in the drafting of elaborate tsetse control and survey plans for implementation under the AU-PATTEC initiative. The methodology can also serve as a template for other PATTEC national initiatives and can be extended to assess the success of vector control programmes.
